# MicroRNA-373 is upregulated and targets TNFAIP1 in human gastric cancer, contributing to tumorigenesis

**DOI:** 10.3892/ol.2013.1534

**Published:** 2013-08-19

**Authors:** XIAOTING ZHANG, XIAOFENG LI, ZHIWEN TAN, XIZHI LIU, CELI YANG, XIAOFENG DING, XIANG HU, JIANLIN ZHOU, SHUANGLIN XIANG, CHANG ZHOU, JIAN ZHANG

**Affiliations:** Key Laboratory of Protein Chemistry and Developmental Biology of State Education Ministry of China, College of Life Science, Hunan Normal University, Changsha, Hunan 410081, P.R. China

**Keywords:** human gastric cancer, miRNA-373, tumor necrosis factor, α-induced protein 1

## Abstract

The role of microRNAs (miRNAs) in regulating gene expression is currently an area of intense interest. Previous studies have shown that miRNA-372 plays crucial roles in gastric tumorigenesis by targeting the mRNA of tumor necrosis factor, α-induced protein 1 (TNFAIP1). The present study showed that miR-373 is upregulated in gastric adenocarcinoma tissue and gastric carcinoma cell lines when compared to normal gastric tissues. The overexpression of miR-373 in the gastric cancer cells increased cell proliferation. A bioinformatics search revealed a conserved target site within the 3′ untranslated region (UTR) of TNFAIP1, an immediate-early response gene of the endothelium induced by TNF-α. The overexpression of miR-373 caused the suppression of a luciferase reporter containing the TNFAIP1 3′UTR in the HEK293 cells and reduced the levels of TNFAIP1 protein in the AGS cells. The mRNA levels of TNFAIP1 in the gastric cancer and normal gastric tissues were negatively correlated with the expression levels of miR-373 in these tissues. Moreover, the knockdown of TNFAIP1 had a similar effect to the overexpression of miR-373. The overexpression of TNFAIP1 may partly rescue the inhibition of proliferation caused by the inhibitor, miR-373-ASO. Taken together, these findings demonstrate an oncogenic role for miR-373, similar to that of miR-372, in controlling cell growth through the downregulation of TNFAIP1.

## Introduction

Gastric cancer is the most frequent type of cancer and remains the second leading cause of cancer mortality worldwide (1). It is estimated that 360,000 individuals succumb to gastric cancer each year in China. Gastric cancer is a heterogeneous disease with various subtypes. In total, >95% of all cancers of the stomach are adenocarcinomas (2). No effective targeting therapy is available for gastric cancer, mainly due to a lack of complete understanding of the molecular mechanisms underlying gastric cancer development (3). Data are now emerging on the potential affect of the disease subtype on the treatment outcome. For example, *HER2* amplification and overexpression is far more prevalent in proximal/gastro-esophageal junction (GEJ) adenocarcinoma than in diffuse gastric cancer. In an exploratory analysis, these proximal/GEJ tumors appeared to be less sensitive to bevacizumab therapy than the diffuse and distal non-diffuse gastric cancers (4). Thus, disease biology may affect patient outcomes with specific treatments.

MicroRNAs (miRNAs) are ~17–24 nucleotides long and act as negative regulators of gene expression by inhibiting mRNA translation or promoting mRNA degradation (5,6). This class of regulators has been described as playing a significant role in a vast range of biological processes, including proliferation, differentiation and apoptosis (7). Recent progress in cancer biology has revealed that miRNAs are frequently deregulated in various types of human cancer, indicating that they may play a role as a novel class of oncogenes or tumor suppressor genes in gastric cancer (8). For example, aberrant miR-106a expression was detected in gastric carcinoma tissues and it was revealed that this may promote gastric carcinogenesis (9). miR-21 has been shown to be correlated with *Helicobacter pylori* infection and gastric cancer progression (10). These data indicate that miRNAs are involved in gastric carcinogenesis via a variety of patterns and pathways and that it may be possible to manipulate miRNA to achieve therapeutic effects.

Previously, it was observed that miR-372 and miR-373 act as oncogenes in the tumorigenesis of human testicular germ cell tumors (Tera-1 and 833KE cells), through the direct inhibition of LATS2 expression (11). In addition, miR-373 was noted to affect esophageal cancer cell growth through the inhibition of LATS2 expression (12). Moreover, we previously reported that miR-372 maintains oncogene characteristics by targeting tumor necrosis factor, α-induced protein 1 (TNFAIP1), and that it regulates AGS cell growth (13). However, it is not clear whether miRNA-373 affects the growth of gastric cancer cells through the inhibition of TNFAIP1 expression. The present study demonstrated that miR-373 is upregulated in gastric adenocarcinoma tissue and gastric carcinoma cell lines when compared to normal gastric tissues. The relative quantification of miR-373 in gastric cancer was 0.5243 times that of normal gastric tissue (P<0.01). Moreover, the bioinformatics prediction and experimental results indicated that miR-373 is able to target TNFAIP1. The study further demonstrated that miR-373 induces the cell proliferation of gastric cancer cells *in vitro*, via the downregulation of TNFAIP1.

## Materials and methods

### Cell culture and transfection

The human gastric carcinoma (AGS, HGC-27 and GES-1) and human embryonic kidney (HEK)293 cell lines were obtained from the Cell Bank of the Chinese Academy of Sciences (Shanghai, China). The cells were cultured in DMEM or F12 media (both from Gibco, Carlsbad, CA, USA) that was supplemented with 10% fetal bovine serum, 100 μg/ml streptomycin and 100 IU/ml penicillin, and maintained at 37°C in a humidified atmosphere with 5% CO_2_. All miRNA mimics and inhibitors that were used in this study were purchased from GenePharma (Shanghai, China). All transfections were carried out using Lipofectamine^®^ 2000 (Invitrogen, Carlsbad, CA, USA), according to the manufacturer’s instructions.

### Human tissue samples

All human gastric adenocarcinoma and adjacent normal tissue samples were obtained from the Department of Pathology at Xiangya Hospital (Changsha, China). The samples were classified according to the World Health Organization criteria that were published in 2000 (14). Informed consent for the use of the samples was obtained from all patients prior to surgery. All patients signed consent forms approved by the Committee on Human Rights in Research of the Ethics Committee at the College of Life Science, Hunan Normal University (Changsha, China).

### RNA extraction and real-time quantification of miRNA-373 and TNFAIP1

Total RNA was extracted using an E.Z.N.A.^®^ FFPE RNA isolation kit (Omega Bio-Tek, Inc., Norcross, GA, USA), according to the manufacturer’s instructions. For miRNA detection, 2 μg small RNA was reverse transcribed to cDNA using an miRNA First-Strand cDNA Synthesis kit (Invitrogen), according to the manufacturer’s instructions. Quantitative (q)PCR analyses for miR-373 and U6 were performed in triplicate with the SYBR-Green PCR Master mix (Perkin-Elmer Applied Biosystems, Foster City, CA, USA), according to the manufacturer’s instructions. U6 RNA was used to normalize expression. To detect the target genes, 2 μg total RNA was reverse transcribed to cDNA using oligo(dT) primers and Moloney murine leukemia virus reverse transcriptase (Promega, Madison, WI, USA). qPCR was used to determine the expression levels of TNFAIP1 and miR-373 using the primers presented in Table I. β-actin levels were used to normalize expression. The data analysis was performed using the 2^−ΔΔCt^ method (15).

### Construction of TNFAIP1 3′ untranslated region (UTR)reporter plasmid

The 3′UTR of TNFAIP1 was amplified from HeLa cDNA using RT-PCR and was inserted into the 3′-end of the firefly luciferase gene of the dual-luciferase miRNA target expression vector pmirGLO (Promega) between the *Sac*I and *Xba*I sites. Equally, the TNFAIP1-3′UTR mutant vectors, which contained mutated miR-373 binding sites, were cloned to the pmirGLO between the same sites. Similarly, the following TNFAIP1 expression vectors were obtained from HeLa cDNA: pCMV-TNFAIP1, which did not contain the 5′ and 3′UTR of TNFAIP1; pCMV-TNFAIP1-3′UTR, which contained the 3′UTR of TNFAIP1; pCMV-TNFAIP1-3′UTRMT, which contained the 3′UTR mutation sites of TNFAIP1. The cells were transfected at 70% confluence using Lipofectamine 2000 transfection reagent, according to the manufacturer’s instruction. The primer sequences used for the RT-PCR amplification are shown in Table I. All primers were synthesized by Sangon Biotech (Shanghai, China).

### Luciferase assay

The dual-luciferase reporter plasmids were co-transfected with miRNA mimics (GenePharma) into HEK293 cells. At 24 h post-transfection, the cells were assayed for luciferase activity using the Dual-Glo Luciferase assay system (Promega), according to the manufacturer’s instructions. The firefly luciferase activities were normalized to Renilla luciferase activity. The firefly luciferase activity of the cells that were transfected with miRNA mimics was represented as the percentage activity relative to that of the cells that were transfected with negative control miRNA mimics. For each transfection, the luciferase activity was averaged from three replicate experiments (16).

### Western blot analysis

At 24 h post-transfection, the cells were harvested and lysed in RIPA buffer [50 mM Tris-HCl (pH 7.2), 150 mM NaCl, 1% (v/v) Triton X-100, 1% (w/v) sodium deoxycholate, 0.1% (w/v) SDS] with protease inhibitors. Proteins were separated on 10% SDS-polyacrylamide gel and transferred to PVDF membranes. A PageRuler prestained protein ladder was used as a molecular marker. Polyclonal TNFAIP1 (ProteinTech Group Inc., Chicago, IL, USA), monoclonal c-myc (Santa Cruz Biotechnology, Santa Cruz, CA, USA) and polyclonal β-actin (endogenous control; CWBIO, Beijing, China) antibodies, were incubated with the blot overnight at 4°C. Goat anti-mouse and goat anti-rabbit secondary antibodies (BA1010 and BA1011; Wuhan Boster Biological Technology, Ltd., Wuhan, China) were added after the membrane was washed three times with TBST. The protein was detected using a HRP-conjugated secondary antibody and a Chemilucent ECL Detection system (Millipore, Billerica, MA, USA).

### MTT assay

Briefly, the AGS or GES-1 cells were seeded in 24-well plates at 16,000 cells/well. At 24 h post-transfection, the cells were incubated with 80 μl MTT at 37°C for another 4 h. The medium was then removed and the precipitated formazan was dissolved in 400 μl DMSO. Subsequent to being shaken for 15 min, the absorbance at 420 nm was detected using a microplate spectrophotometer.

### Statistical analysis

All the results are presented as the mean ± standard deviation from at least three separate experiments. The differences among groups were analyzed using the double-sided Student’s t-test, and P<0.05 was used to indicate a statistically significant difference.

## Results

### miR-373 is upregulated in human gastric adenocarcinoma tissue and AGS cells

**Figure 1:**
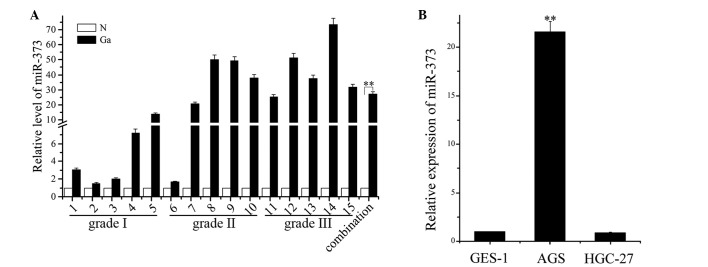
miR-373 was upregulated in gastric adenocarcinoma tissues and cell lines compared to normal gastric tissues. (A) RT-PCR analysis of miR-373 in 15 pairs of gastric adenocarcinoma tissues (Ga; 1–5, grade I; 6–10, grade II; and 11–15, grade III) and matched adjacent normal tissues (N). ^**^P<0.01 vs. adjacent normal tissues. (B) RT-PCR analysis of miR-373 in human gastric carcinoma cell lines (AGS, HGC-27 and GES-1). U6 snRNA was used as an endogenous normalizer, and the relative expression levels of miR-373 from the 15 pairs of gastric tissues and the three types of cells, as well as the combined results, are shown. The data represent the mean ± SD of three different experiments. ^**^P<0.01 vs. GES-1.

To confirm the expression level of miR-373 in gastric adenocarcinoma tissue and normal gastric tissues, total RNA was extracted from 15 gastric adenocarcinoma samples, consisting of five grade I, five grade II and five grade III gastric adenocarcinoma tissues, 15 matched normal tissues and three human gastric carcinoma cell lines, and then qPCR was performed to analyze the expression profile. Abnormal upregulation of miR-373 was observed in the gastric adenocarcinoma tissue and AGS cells, while low expression was observed in the matched normal tissue, indicating that miR-373 expression is upregulated in gastric cancer (Fig. 1A). The relative quantification of miR-373 in gastric cancer was 26.861 times that of miR-373 in the normal gastric tissue (P<0.05). Previously, Cho *et al*(17) analyzed the expression of the miRNA-371–373 cluster in four human gastric cancer cell lines, SNU-1, SNU-638, SNU-719 and AGS, and one normal human gastric cell line, Hs677.sT, and identified that miR-372 expression was markedly elevated in the AGS cells. The present study has shown the expression of miR-373 in three gastric cancer cell lines to be consistent with a previous study (16) (Fig. 1B), indicating that this may play a possible role as an oncogene.

### miR-373 negatively regulates TNFAIP1 expression by targeting the 3′UTR

**Figure 2:**
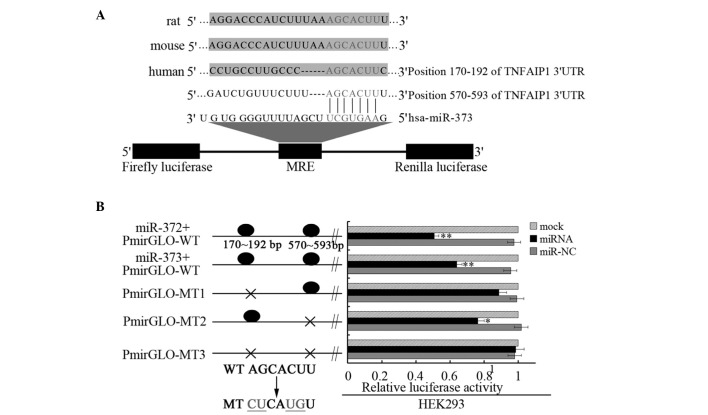
Tumor necrosis factor, α-induced protein 1 (TNFAIP1) is one of the direct targets of miR-373. (A) Diagram of luciferase reporter constructs and the binding site of miR-373 in the predicted target sequences. (B) Sketch of TNFAIP1 mutated vectors and luciferase assay in HEK293 cells. HEK293 cells were transiently co-transfected with luciferase reporter constructs with miR-373 or negative control miRNA. Cells transfected with empty luciferase vector were used as a mock control. The mutated site of the TNFAIP1 3′ untranslated region (UTR) is indicated in underlined italics. The relative luciferase activities were normalized against the Renilla luciferase activities. The data represent the mean ± SD of three different experiments. ^*^P<0.05; ^**^P<0.01 vs. mock.

Two putative miR-373 binding sites were predicted to have greater specificity to the TNFAIP1 3′UTR, ranging from dinucleotide 170–192 bp or 570–593 bp, as predicted by four algorithms (TargetScan, PicTar, miRanda and miRBase Target). Moreover, this putative miRNA response element (MRE) was highly conserved in vertebrates (Fig. 2A). The present study validated whether the predicted MRE could be recognized by the miR-372/miR-373 family using the dual-luciferase vector pmirGLO. The predicted MRE, wild-type 3′UTR of TNFAIP1, was cloned downstream of the firefly luciferase of the pmirGLO vector and co-transfected with miR-372 or miR-373 mimics (double-stranded processed miRNA) into HEK293 cells, which do not express miR-372 or miR-373. The expression of miR-372 or miR-373 suppressed the firefly luciferase activities of the MRE-containing 3′UTR of TNFAIP1 (Fig. 2B). Three mutant vectors of the 3′UTR of TNFAIP1 were also constructed: Two single site mutation vectors, with one binding site with miR-373 and a whole site mutation vector, with no binding site with miR-373. Then, a luciferase reporter assay was performed with the mutated constructs in the HEK293 cells. Once the conserved targeting regions for miR-373 recognition had been mutated, the relative luciferase activity of the TNFAIP1 gene was also restored. The results demonstrated that the luciferase activity was elevated in all the mutated vectors and that the relative luciferase activity of the two single site mutation vectors increased by a lower percentage compared with the whole site mutation vector. These observations indicate that the predicted complementary sequence in TNFAIP1 3′UTR is a functional element of miR-373.

### TNFAIP1 is downregulated in human gastric adenocarcinoma tissue

**Figure 3:**
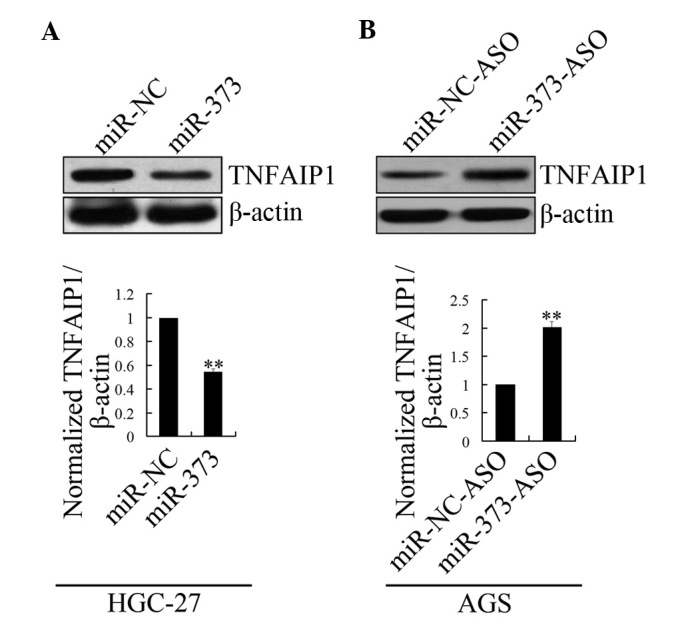
Quantitative analysis of tumor necrosis factor, α-induced protein 1 (TNFAIP1) expression in human gastric adenocarcinoma tissues. (A) The expression levels of TNFAIP1 were measured by quantitative (q)PCR. The 15 pairs of gastric adenocarcinoma tissues (Ga) and matched adjacent normal tissues (N) from Fig. 1 were used again to measure the relative TNFAIP1 expression levels. β-actin was used as an endogenous normalizer. (B) The coefficients of correlation between miR-373 and TNFAIP1 in human gastric adenocarcinoma tissues. Data represent the average of three independent runs; bars, mean ± standard error (SEM). ^**^P<0.01.

The high expression of miR-373 was observed in human gastric adenocarcinoma tissue and AGS cells, while the potential expression levels of TNFAIP1 were not clear. Although TNFAIP1 has been widely reported to be involved in tumorigenesis, there are no studies on its role in human gastric adenocarcinoma tissue. To investigate whether miR-373 affects the TNFAIP1 expression *in vivo*, the mRNA levels of TNFAIP1 in gastric adenocarcinoma tissue and normal gastric tissues were determined by RT-PCR. A correlation was observed between the level of miR-373 and the mRNA levels of TNFAIP1 in gastric adenocarcinoma tissue. This showed that, in grades I, II and III gastric adenocarcinoma tissues, the upregulation of miR-373 was more marked than that in 15 matched normal tissues, while the mRNA levels of TNFAIP1 in all investigated gastric adenocarcinoma tissues were lower than those in matched normal tissues (Fig. 3A). High expression of miR-373 inversely correlated with lower expression of TNFAIP1 in human gastric cancer (Fig. 3B). In conclusion, these results showed an inverse correlation between miR-373 and TNFAIP1 mRNA levels.

### miR-373 represses endogenous TNFAIP1 expression

**Figure 4:**
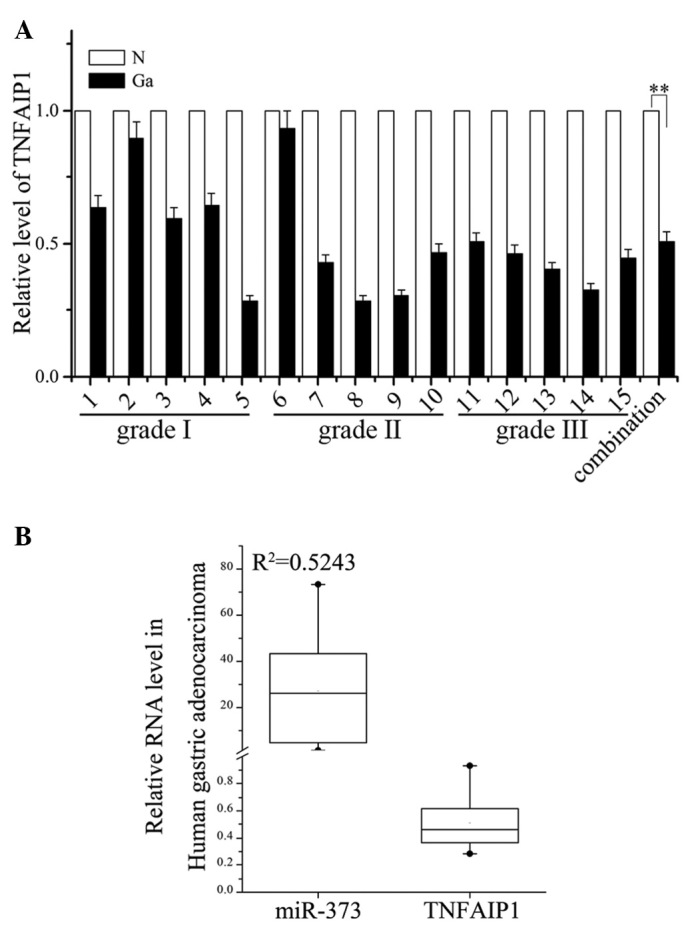
Tumor necrosis factor, α-induced protein 1 (TNFAIP1) expression is negatively regulated by miR-373 in gastric adenocarcinoma cells. (A) HGC-27 cells were transfected with miR-373 or miR-NC, whereas (B) AGS cells were transfected with miR-373-ASO or miR-NC-ASO, respectively. After 48 h, cell lysates and western blot analysis were used to detect the expression of TNFAIP1. Data represent the average of three independent runs; bars, mean ± standard error (SEM). ^**^P<0.01 vs. control.

To examine the effect of miR-373 on endogenous TNFAIP1 expression, miR-373 mimics were transfected into HGC-27 cells, which are known to express high levels of TNFAIP1 protein. The enhanced expression of miR-373 in the HGC-27 cells significantly decreased the amount of TNFAIP1 protein expression compared with mock transfection (Fig. 4A). However, when transected with miR-373 inhibitors (miR-373-ASO) in AGS cells, which are 2′OMe chemically modified, single-stranded nucleic acids designed to specifically bind to and inhibit endogenous miR-373 molecules, the observations indicated that TNFAIP1 was negatively regulated by miR-373 at the protein level (Fig. 4B).

### miR-373 regulates growth of gastric cell lines

**Figure 5:**
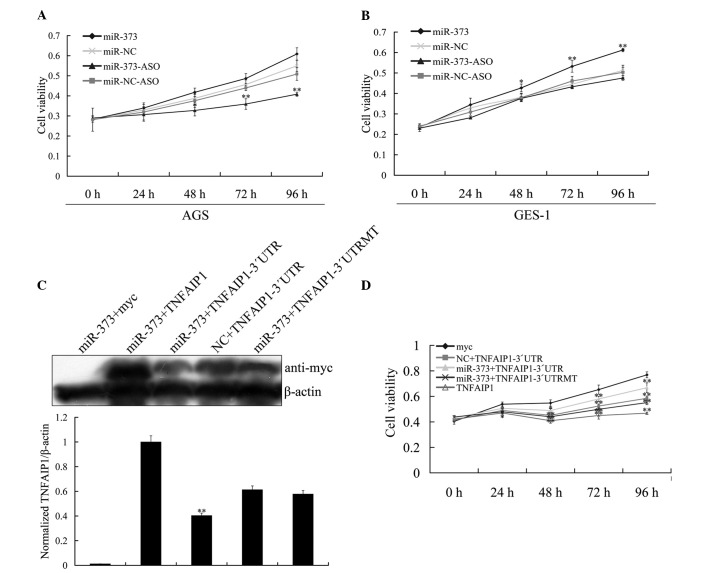
miR-373 regulates growth of AGS and GES-1 gastric cell lines. MTT assay of (A) AGS or (B) GES-1 cell proliferation from 0 h to 96 h following transient transfection with miR-373, miR-NC, miR-373 inhibitor (miR-373-ASO) and miR-NC-ASO. (C) Western blotting for the transfection of AGS cells with three tumor necrosis factor, α-induced protein 1 (TNFAIP1) expression vectors and miR-373 or miR-NC. (D) Cell growth was determined by MTT assay every 24 h. The data represented the average of three independent runs; bars, SEM. ^*^P<0.05; ^**^P<0.01.

Gain-of-function experiments are widely used for functional studies of miRNAs. To further investigate whether the correlation between miR-373 and gastric cell lines only existed in gastric cancer cells, GES-1 cells, which are normal human gastric mucosal cells, were introduced into the experiment. The relative expression of miR-373 measured using RT-PCR demonstrated that miR-373 expression levels in the AGS cells were markedly higher than in the GES-1 cells, where the level was almost undetected. The growth of gastric cell lines affected by miR-373 was then studied using an MTT assay. To characterize the effects of miR-373 on cell growth, the AGS cells were transfected with miR-373-ASO, and growth was monitored using a cell growth curve, which indicated that the knockdown of miR-373 inhibited cell growth. The growth rate of the AGS cells transfected with the miR-373 inhibitor was lower than in those transfected with the miR-NC, miR-NC-ASO and miR-373 mimics (Fig. 5A). To determine whether increasing miR-373 reversed the inhibition of cellular proliferation observed due to overexpression, the GES-1 cells were transfected with miR-373 mimics and compared with the results for miR-373-ASO, miR-NC-ASO and miR-NC. An increase in proliferation was observed in the cells transfected with miR-373 mimics as compared with cells transfected with the control (Fig. 5B). These studies indicate that AGS and GES-1 cell growth may be positively modulated by miR-373.

### miR-373 induces AGS cell proliferation by targeting TNFAIP1

In order to further investigate the effect of miR-373 on the regulation of TNFAIP1 expression in the AGS cells, AGS cells grown on a 6 cm plate were co-transfected with miR-373 mimics and TNFAIP1 expression vector pCMV-TNFAIP1, pCMV-TNFAIP1-3′UTR and pCMV-TNFAIP1-3′UTRMT, respectively. Western blotting assays revealed an inverse correlation between miR-373 and TNFAIP1 protein levels (Fig. 5C). MTT assays showed that the proliferation rate was reduced following the overexpression of TNFAIP1, which was able to produce the same effect as miR-373 inhibitor treatment in the AGS cells (Fig. 5A and D). This supports the hypothesis that miR-373 induces the proliferation of gastric cancer cell lines by negatively regulating the expression of TNFAIP1 at the protein level.

## Discussion

Documented evidence has demonstrated that miRNAs may function as a novel class of tumorigenetic and tumor suppressing genes. A more direct link between miRNA function and oncogenesis is supported by studies examining the expression of miRNAs in clinical samples (18–21). For example, the profiling of miRNA expression showed that the majority of miRNAs are downregulated in tumors compared to normal tissues, such as miR-128 in glioma tissues (22) and miR-145 in human breast cancer (23). However, miR-17–92 is significantly increased in small-cell lung cancers and human B-cell lymphomas and plays a key role in tumorigenesis (24). Recent evidence suggests that miR-373 is tumorigenetic in human reproductive system cancers and human embryonic stem cells (ESCs) by targeting the tumor suppressor LATS2 (25). In the present study, the expression of miR-373 was detected in human gastric adenocarcinoma tissue samples and gastric cancer cell lines and observed to be upregulated compared to levels in normal gastric tissues. This conclusion was established by results showing that the downregulation of miR-373 expression by miR-373-ASO suppressed the growth of AGS cells and increased apoptosis. Overexpression of miR-373 in the HGC-27 cells increased cell growth. These findings are in agreement with a previous report demonstrating that miR-373 has oncogenic properties (11).

Although a large number of miRNAs have been discovered, only a few targets have been identified. The identification of miRNA target genes remains a great challenge. Computational algorithms showed >500 targets of miR-373. Considering that miR-373 overexpression is able to increase the proliferation of gastric cancer cells, several predicted target genes associated with tumorigenesis or cell proliferation were subjected to a luciferase reporter assay in the present study. The results showed that, among these selected genes, TNFAIP1 was negatively regulated by miR-373. These findings are in agreement with a previous study demonstrating that miR-372 targets TNFAIP1. To determine whether miR-373 has a direct effect on TNFAIP1 expression, miR-373 mimics or miR-373-ASO were transfected into the HGC-27 or AGS cells to alter the level of miR-373. When the AGS cells were transfected with miR-373 inhibitors (miR-373-ASO), which are 2′OMe chemically modified, single-stranded nucleic acids designed to specifically bind to and inhibit endogenous miR-373 molecules, the observations indicated that TNFAIP1 was negatively regulated by miR-373 at the protein and mRNA levels. Conversely, treatment with miR-373 mimics or control (miR-NC) was performed in the HGC-27 cells. The results showed that the overexpression of miR-373 markedly decreased the expression of TNFAIP1 at the protein and mRNA levels. These observations indicate that TNFAIP1 is negatively regulated by miR-373.

The expression levels of TNFAIP1 were measured in 15 pairs of human gastric adenocarcinoma tissue and adjacent normal tissues using RT-PCR. The results showed that TNFAIP1 expression levels were generally lower in the human gastric adenocarcinoma tissues than in the matched normal gastric tissues, indicating that TNFAIP1 expression is downregulated in gastric cancer. One notable point observed in this study was that the upregulation of miR-373 and the downregulation of TNFAIP1 was all in grade I, II and III gastric adenocarcinoma tissues. Moreover, a high expression of miR-373 inversely correlated with a lower expression of TNFAIP1 in these gastric adenocarcinoma tissues. Gastric cancer is a heterogeneous disease, with subtypes in existence. There is, however, a limitation within the present data interpretation with regard to the correlated expression of miR-373 and TNFAIP1, since human gastric adenocarcinoma tissue and normal gastric tissues were used to detect their expression levels. Nevertheless, the stomach is a mixture of different cell types and detection of miR-373 and TNFAIP1 by other methods may be required. Whether miR-373 and TNFAIP1 may be used as molecular markers for the diagnosis of gastric adenocarcinoma requires more evidence.

TNFAIP1, also termed B12, is highly conserved in a wide range of species, including humans, mice, rats (26) and *C. elegans*(27). A high expression of TNFAIP1 has been observed in normal cell lines, while a low level of expression has been detected in cancer cell lines (28). TNFAIP1 is an immediate-early response gene of the endothelium induced by TNF-α and IL-6 (29). TNFAIP1 may play roles in DNA synthesis, DNA repair, cell apoptosis and human diseases (30). A previous study (28) demonstrated that the TNFAIP1 gene was weakly expressed in several cancer-derived cell lines, while it was expressed at a high level in normal cells and also modulated the cellular proliferation rate. Conversely, reducing miR-373 expression using miRNA inhibitors greatly sensitized the cells to apoptosis. miR-373 overexpression affected the cell growth and correlated with the level of TNFAIP1. Thus, we conclude that miRNA-373 accelerates gastric cancer cell differentiation by negatively regulating TNFAIP1.

In conclusion, the present study established the important role played by miR-373 in gastric cancer. Following the observation of the elevated expression of miR-373 in human gastric adenocarcinoma tissue samples and AGS cells, the study demonstrated that: i) Overexpression of miR-373 is associated with accelerated cell differentiation; ii) miR-373 directly targets and downregulates the expression of TNFAIP1; and iii) the knockdown of miR-373 by miRNA inhibitors recapitulates the differentiation phenotype created by the overexpression of TNFAIP1. Collectively, these findings demonstrate an oncogenic role for miR-373 in controlling cell growth and apoptosis through the downregulation of TNFAIP1.

## Figures and Tables

**Table I:** Oligonucleotides used in the study.

Name	Sequence (5′→3′)
pmirGLO-TNFAIP1	
Forward	CGAGCTCGTGCTGCCTGGGTCTCTGC
Reverse	GCTCTAGAGCAGCTGCTCTGTCGGATGTTT
pmirGLO-TNFAIP1-mut1	
Forward	CCTGCTCATGTCTGGAGAC
Reverse	CAGACATGAGCAGGGGCAA
pmirGLO-TNFAIP1-F2	TGTGCAGAAGGGCTACTGC
pmirGLO-TNFAIP1-R2	GCAGTAGCCCTTCTGCACA
pmirGLO-TNFAIP1-mut2	
Forward	ATTCTCATGTACATGACAATAAG
Reverse	TCAGTACAACATGAGTTAAAGAA
PCMV-TNFAIP1-CDS	
Forward	AGTCGACGATGTCAGGGGACACCTGT
Reverse	GGGTACCTCAGTCACGATGAGTGGA
PCMV-TNFAIP1-3′UTR	
Forward	AGTCGACGATGTCAGGGGACACCTGT
Reverse	GCTCTAGAGCAGCTGCTCTGTCGGATGTTT
pCMV-TNFAIP1-3′UTRMT1	
Forward	CCTGCTCATGTCTGGAGAC
Reverse	CAGACATGAGCAGGGGCAA
pCMV-TNFAIP1-3′UTRMT2	
Forward	ATTCTCATGTACATGACAATAAG
Reverse	TCAGTACAACATGAGTTAAAGAA
RT-TNFAIP1	
Forward	GCACTTTGGGCACCATTTTGA
Reverse	CGGTTCTGAGGGAGGGTGAT
U6	TGCGGGTGCTCGCTTCGGCAGC
β-actin	
Forward	CCTGTACGCCAACACAGTGC
Reverse	ATACTCCTGCTTGCTGATCC

Restriction or mutation sites are underlined. TNFAIP1, tumor necrosis factor, α-induced protein 1.
